# Authors’ reply to Hansel’s letter to the editor

**DOI:** 10.1186/s13054-023-04510-w

**Published:** 2023-06-08

**Authors:** Yuki Kotani, Alessandro Pruna, Todd C. Lee, Giovanni Landoni

**Affiliations:** 1 Department of Anesthesia and Intensive Care, IRCCS San Raffaele Scientific Institute, Via Olgettina 60, 20132 Milan, Italy; 2 School of Medicine, Vita-Salute San Raffaele University, Via Olgettina 58, 20132 Milan, Italy; 3 Department of Intensive Care Medicine, Kameda Medical Center, 929 Higashi-Cho, Kamogawa, Chiba 296-8602 Japan; 4 Division of Infectious Diseases, Department of Medicine, McGill University, Montreal, QC Canada

Safety concerns surrounding propofol date back beyond 2001 when the first US Food and Drug Administration warning was issued [1]. Our previous meta-analysis [2] suggested a 10% increase in mortality when comparing propofol (5.0%) vs. any comparator (4.5%) in any setting although this did not meet statistical significance with 133 randomized trials comprising 14,156 subjects. On this background, we set out to update the meta-analysis. We chose to compare propofol to all other agents to determine if there was a relative harm signal related to this agent. A similar “all comparators and settings” approach led to the Food and Drug Administration issuing a warning against tigecycline [3].

In performing our analysis, we attempted to be as inclusive as possible and so extracted mortality at the longest follow up available. Variations in follow-up time have been described in critical care settings and in meta-regression, these were not found to influence pooled point estimates of the effects on mortality [4]. It was suggested that pooling mortality data from different time points can reasonably improve the precision of the pooled effect estimate. In our meta-analysis, cumulative and trial sequential analysis techniques show that this effect is constant over time and suggest that statistical significance is approaching as data accumulates.

We extracted data following the intention-to-treat strategy. Nonetheless, we acknowledge that the patients missing from each group in the 1-year follow-up of the Likhvantsev study would have to be assumed to have survived, and that may not be the case. We repeated the cardiovascular subgroup analysis following different extraction approaches (Additional file 1: Table S1).

We used the Mantel–Haenszel method because it is preferred in the Cochrane manual and a fixed-effects model given the very low statistical heterogeneity [5]. We explored clinical heterogeneity performing multiple subgroup analyses (reported in the supplement and summarized in the main manuscript), which confirmed the magnitude and direction of the detrimental effect of propofol on survival in all settings.

We now report the overall analyses also using the random-effects model and mortality data at the closest time point to 30 days (Additional file 1: Table S1).

There remains a sizable signal of harm that warrants further prospective study. Thousands of patients are receiving propofol in a variety of settings every day. It is time to challenge the status quo and conduct large multicentered randomized controlled trials in different care settings designed to evaluate the safety of propofol based sedation.

## Supplementary Information

**Additional file 1.** Supplemental Table 1

## Data Availability

Further information on the original manuscript are available from the corresponding authors upon reasonable request.
